# Comparative analysis of various step-dilution techniques on the quality of frozen Limousin bull semen

**DOI:** 10.14202/vetworld.2020.2422-2428

**Published:** 2020-11-13

**Authors:** Ani Atul Arif, Tulus Maulana, Ekayanti Mulyawati Kaiin, Bambang Purwantara, Raden Iis Arifiantini, Erdogan Memili

**Affiliations:** 1 Reproductive Biology Study Program, Faculty of Veterinary Medicine, IPB University, Bogor, Indonesia; 2 Biotechnology Livestock Research Group, Research Center for Biotechnology, Indonesian Institute of Science, Bogor, Indonesia; 3 Department of Veterinary Clinics, Reproduction and Pathology, Division of Veterinary Reproduction and Obstetrics, Faculty of Veterinary Medicine, IPB University, Bogor, Indonesia; 4 Department of Animal and Dairy Sciences, Mississippi State University, Starkville, MS, United States

**Keywords:** aminotransferase, dilution technique, frozen semen quality, malondialdehyde

## Abstract

**Background and Aim:**

Indonesia has two National Artificial Insemination centers and 17 Regional Artificial Insemination Centers. The frozen semen production techniques differed between the centers, including the type of diluent and semen dilution technique. The aim of the research was to compare the quality of frozen Limousin bull semen diluted using different techniques.

**Materials and Methods:**

Semen was collected from three sexually mature Limousin bulls using an artificial vagina. Immediately after collection, the semen was evaluated macroscopically and microscopically. Semen that had >70% motile sperm and <20% sperm abnormality was divided into three tubes and diluted with skim milk-egg yolk (SMEY) using three different dilution techniques: One-step dilution (100% SMEY with 8% glycerol) at room temperature ([RT] 20°C until 25°C) two-step dilution (50% SMEY without glycerol at RT, stored at 5°C; and 50% SMEY with 16% glycerol after 1 h stored at 5°C); and three-step dilution (50% SMEY without glycerol at RT, stored at 5°C; and 50% SMEY with 16% glycerol added twice at 1 h and 1.5 h after being stored at 5°C). The diluted semen was loaded into 0.25 mL mini straws, equilibrated, and frozen using a freezing machine. Sperm motility, viability, membranes, DNA integrity, and concentrations of malondialdehyde (MDA) and aspartate aminotransferase (AST) enzymes were evaluated after thawing.

**Results:**

The results showed that there were no significant differences in sperm motility and DNA integrity between dilutions (p*>*0.05). However, sperm viability and membrane intactness of one-step dilutions were higher than those of three-step dilutions. The concentrations of MDA and AST enzymes of sperm in one-step dilutions were lower than those of three-step dilutions (p*<*0.05).

**Conclusion:**

It was concluded that the one-step-dilution technique was better than three-step dilution for cryopreservation of Limousin bull semen.

## Introduction

Indonesia has two National Artificial Insemina- tion Centers (AI Centers), namely, the Singosari and Lembang AI Centers. Indonesia also has several Regional AI Centers in various provinces; including Aceh, North Sumatra, West Sumatra, South Sumatra, Lampung, West Java, Central Java, Yogyakarta, South Kalimantan, East Kalimantan, Southeast Sulawesi, South Sulawesi, West Nusa Tenggara, and Bali [1]. The main task of the AI Centers is to produce good quality frozen (cryopreserved) semen from superior bulls, such as the Limousin bull. The Limousin bull is one of the most popular exotic bulls for the AI program because exotic calves have a high value; thus, there is an increasing demand for frozen semen of these bulls [2]. Success in the semen freezing process is influenced by the type of diluents [3] as well as the dilution and freezing techniques used [4]. The process of frozen semen production requires an extender to maintain the quality of semen. One of the most common extenders used for this purpose is skim milk-egg yolk extender (SMEY) [5]. The processes of adding extenders to semen vary. Some AI Centers add an extender to the semen only once at room temperature ([RT] 20°C until 25°C) (one-step dilution), other AI Centers use two-step dilutions, but there are also AI Centers that use three- or four-step dilutions.

The merits of one-step, two-step, and gradual semen dilution techniques are still being debated. The one-step-dilution method is more practical because the extender is mixed with semen at RT before being packed, equilibrated, and frozen [6]. The two-step semen dilution method includes using the first extender without glycerol, followed by the addition of a second extender containing glycerol, which is usually done at 4°C-5°C before it is equilibrated [6] and packaged at 4°C-5°C. Two-step dilution has been shown to reduce the quality of boar semen [7]; whereas for dog semen, a two-step dilution is better than a one-step dilution [4]. In dilutions with more than two steps, addition of a glycerol extender is carried out several times at 4°C-5°C. The gradual dilution technique causes repeated temperature changes [8] as a result of opening and closing the cooling cabinet in which the semen is stored. Addition of glycerol at 4°C-5°C yields better results compared to the addition of glycerol at RT. Cell membranes are less permeable to glycerol at 4°C-5°C; thus, the toxic effects of glycerol are reduced [3]. The semen cryopreservation process generally causes physical damage to the plasma membrane and mitochondria, and molecular changes such as DNA fragmentation, mRNA degradation, chromatin changes, and generation of reactive oxygen species (ROS). The latter is characterized by the presence of malondialdehyde (MDA) and aspartate aminotransferase (AST) enzymes. Thus, the freezing process generally decreases the overall quality of semen, regardless of the technique used [9].

The different dilution techniques used by Indonesian AI Centers have not been scientifically compared; therefore, it is necessary to evaluate and determine the most appropriate and practical dilution techniques in an effort to improve the quality of frozen semen, especially of Limousin bulls.

This study aimed to evaluate and compare the effects of multiple dilution techniques on the post-thaw quality of frozen semen from Limousin bulls, using a skim milk diluent.

## Materials and Methods

### Ethical approval

This research was carried out following standard operational procedure SNI ISO 9001:2015 No. 824 100 15084 at the Ungaran AI Center in Central Java, supervised by a veterinarian from this institution. The Ethical Committee of Ungaran AI Center in Central Java provided ethical guidelines on the responsible use of bulls for semen collection and approved this experiment.

### Study period and location

This research was conducted from September 2019 to January 2020 in the Laboratory of Semen Processing at Ungaran Regional AI Center-Central Java-Indonesia, Laboratory of Animal Cell Reproduction-Breeding and Culture-Biotechnology Livestock Research Center-Indonesian Institute of Sciences (LIPI)-Cibinong, Laboratory of Reproductive Rehabilitation Unit-Faculty of Veterinary Medicine and Laboratory Biochemistry-Faculty of Mathematics and Natural Sciences- IPB University-Bogor-West Java-Indonesia.

### Sampling and semen processing

Three Limousin bulls, each 3 years of age, were used as sources of semen. Four ejaculates were collected from each bull. Only semen ejaculates with more than 70% sperm motility and <20% sperm abnormalities were used. All bulls were kept in individual pens that were equipped with a place to feed and drink. Feeding included forages and concentrates, as much as 10% and 1% of the body weight, respectively. The feed was given in the morning and afternoon. Drinking water was provided ad libitum. Semen was collected using an artificial vagina twice a week, in the morning, according to the standard protocol of the AI Center. Immediately after collection, the fresh semen was taken to the laboratory for macroscopic and microscopic evaluations [10]. The macroscopic evaluation included volume, color, consistency, and pH. The microscopic evaluation included mass movement, motility, viability, sperm concentration, and morphology.

The extender used was SMEY. SMEY extender was divided into two parts, namely, Part A and Part B. Part A was a semen extender consisting of 90% antibiotic skim buffer containing 10 g skim milk, 100 mL distilled water, 1000 IU/mL penicillin, 1 mg/mL streptomycin, and 10% egg yolk from the total extender volume. Part B was a Part A extender that had been supplemented with 16% glycerol (Merck, Germany) and 2 g of fructose (Merck, Germany). Fresh semen with >70% sperm motility and <20% sperm abnormalities was divided into three tubes. Each of them was diluted using three dilution techniques, namely, one-step, two-step, and three-step dilutions.

#### One-step dilution

Semen was combined with 50% Part A and 50% Part B extenders (100×10^6^/mL sperm) at RT, homogenized, loaded into a 0.25 mL mini straw (IMV, France), and equilibrated in a cooling cabinet for 4 h. The semen mixture was frozen using a freezing machine (Minitube, Germany). The frozen semen was stored in liquid nitrogen for further evaluation.

#### Two-step dilution

Semen was diluted with 50% of the total dilution of Part A extender at RT. Diluted semen was stored in a cooling cabinet. Part B extender was stored in the same cooling cabinet. A 50% Part B extender (i.e., the same amount as Part A) was added 1 h later and the mixture was left to equilibrate for 4 h. Then, the semen was loaded into a cooling cabinet and frozen, as in the one-step dilution.

#### Three-step dilution

Semen was diluted by 50% with Part A extender. The addition of Part B extender was carried out twice, that is, 25% each, 30 min apart. Semen was equilibrated at 5°C for 4 h. The packaging and freezing processes were carried out using the same protocols as in the one- and two-step dilutions.

All frozen semen samples were stored in liquid nitrogen for further evaluation. Semen quality evaluation was conducted by thawing the semen in the straw in warm water (37°C) for 30 s. The following quality evaluations were carried out: Sperm motility, viability, plasma membrane integrity (PMI), DNA damage, MDA level, and AST enzyme concentration.

### Sperm motility

Sperm motility analysis was conducted objectively using computer-assisted sperm analysis (CASA, Sperm Vision 3.7; Minitube, Germany). Ten microliters of the thawed semen were dripped onto a glass slide and covered with a glass cover. Sperm motility was assessed using CASA, which is based on the analysis of digitalized images from a computer connected to a microscope at 200×. The evaluations were carried out in four visual fields [11].

### Sperm viability

Evaluation of sperm viability was performed using eosin-nigrosin staining. A total of 50 μL of thawed semen was dripped onto a glass slide before 100 μL of eosin-nigrosin was added. The mixture was then homogenized, smeared on an object slide, and dried over a heating table. Observations were carried out using a microscope at a magnification of 400× in ten fields of view or 200 cells. The living sperm showed up clear-colored (transparent) and dead sperm absorbed the red color on the head.

### Integrity of the sperm plasma membrane

Evaluation of the integrity of the sperm plasma membrane was conducted using a hypoosmotic swelling (HOS) solution test. A total of 50 μL of thawed semen was added to a microtube containing 950 μL of HOS solution before the mixture was homogenized and incubated at 37°C for 30 min. Thereafter, 10 μL of the sample was dripped onto a glass slide and covered with a glass cover for observation using a microscope at 400× in ten fields of view or 200 cells. Sperm with an intact plasma membrane had a coiled tail.

### Sperm DNA damage

Sperm DNA damage was determined using Acridine Orange (AO) staining. Twenty microliters of thawed semen were prepared as a sperm smear preparation, then dried and fixed in Carnoy’s solution (1:3 mixture of acetic acid and methanol) for 4 h. Then, the sample was fixed and rinsed using distilled water before being dried and immersed in an AO solution in a dark room for 12 h. The sample was air-dried at RT in a dark room for 5 min. The samples were analyzed using a fluorescence microscope with a magnification of 400× in a dark room [12]. Sperm cells with intact DNA were colored green, while sperm cells with damaged DNA were a yellow to orange color. These evaluations were carried out on 500 sperm cells from each sample.

### Sperm MDA level

Evaluation of sperm MDA levels was carried out using the thiobarbituric acid (TBA) method [13]. One milliliter of thawed semen was centrifuged (Kitman-T24, Tommy, Japan) at 1000× *g* for 10 min. The supernatant was removed, and the sperm pellet was washed 2 times with Tris-HCl pH 7. The pellet was added to 1 mL distilled water and 0.5 mL TBA reagent (0.67 g 2-TBA, 100 mL distilled water, 0.5 g NaOH, and 100 mL glacial acetic acid). The sample was heated at 90°C for 1 h and centrifuged at 4000× *g* for 10 min. The absorption of the resulting pink supernatant was measured using a spectrophotometer at a wavelength of 532 nm. The MDA coefficient used was 1.56×10^5^/M/cm, and the results were expressed in MDA/10^8^ sperm counts.

### Sperm AST concentration

The AST enzyme concentration was analyzed using a commercial kit (Glory Science Co., Ltd., Spain). One milliliter of thawed semen was centrifuged at 2500× *g* for 10 min. Fifty microliter supernatant was added to 1 mL of the commercial reagent kit, homogenized, and incubated at 37°C for 1 min. The absorbances after 1, 2, and 3 min were calculated using readings taken at a wavelength of 340 nm. The AST concentration was calculated using the formula U/L=ΔA/min × 3333.

### Statistical analysis

This study used a complete randomized design with four replications. The data obtained were analyzed using analysis of variance at a 95% significance level. If there were significant differences between treatments, further analysis was performed using Duncan’s Multiple Range Test. The data were analyzed using SPSS Version 24.0 (IBM Corp., NY, USA), and presented as mean±standard error (SE).

## Results and Discussion

### Fresh semen quality

The quality of fresh semen in the study was good, with an average volume of 6.23±0.24 mL and pH 6.48±0.04. The semen color was milky white to creamy with a moderate to thick consistency. Sperm mass movement was high (+++) with >70% sperm motility. Sperm concentration was 1196±53.31×10^6^/mL. Sperm viability was 94.07±0.76%, with low sperm abnormality (2.35±0.26%), and intact plasma membrane occurrence of 92.58±0.56%. The quality of fresh semen from Limousin bulls is in accordance with results reported by Wiratri *et al*. [14]. Sperm motility of fresh semen to be processed into frozen semen meets the quality requirements of the Indonesian National Standards on frozen semen from bulls [15]. The results of macroscopic and microscopic evaluations of Limousin bulls’ fresh semen showed high quality and that they could be processed into frozen semen.

### Sperm motility and kinematics in frozen semen of Limousin cattle using CASA on various dilution techniques

One-, two-, and three-step-dilution techniques showed no significant difference in sperm motility and kinematics (p>0.05). Total sperm motility ranged from 53.93±5.81% to 64.25±2.84%. Progressive sperm motility ranged from 41.12±15.19% to 53.67±3.55%. Some parameters of sperm kinematics relate to fertility [16], including curvilinear velocity (VCL), average pathway velocity (VAP), straight line velocity (VSL), beat cross frequency (BCF), and amplitude of lateral head displacement (ALH). The VCL, VAP, and VSL values needed for sperm to be able to penetrate the ovum are VCL >70 μm/s, VAP, and VSL >45 μm/s [17]. BCF values >20 Hz and ALH values between 2.5 and 6.5 μm indicate that sperm movement is optimal and has good fertility [18].

The results of this study showed that the VCL, VAP, and VSL values of all diluted sperm (i.e., irrespective of which dilution technique was used) were higher than the minimum requirements for fertilization. The BCF values of all dilution techniques ranged from 20.45±0.82 to 23.33±2.11 Hz. The ALH values were between 4.40±0.57 and 5.50±0.79 μm (Table-1). From these results, it can be extrapolated that dilution technique does not affect the ability of sperm to fertilize an ovum. It can also be concluded that SMEY extender could protect sperm during freezing and thawing.

**Table-1:** Sperm kinematics of Limousin bull frozen semen in the skim milk-egg yolk extender on various dilution techniques.

Sperm kinematic parameters	Dilution techniques		

	One-step	Two-steps	Three-steps
Total motility (%)	64.25±2.84	60.27±3.72	53.93±5.81
Progressive motility (%)	53.67±3.55	48.73±2.46	41.12±15.19
Velocity curvilinear (µm/s)	120.57±4.03	109.42±18.12	107.51±14.30
Velocity average path (µm/s)	75.50±3.00	71.24±9.83	63.15±7.12
Velocity straight line (µm/s)	59.96±5.63	58.37±6.56	48.38±2.96
Beat cross frequency (Hz)	23.33±2.11	23.83±2.25	20.45±0.82
Amplitude of lateral head displacement (µm)	4.99±0.12	4.40±0.57	5.50±0.79

Sperm motility is a parameter commonly used to assess semen quality. Progressive motility of all dilution techniques has fulfilled the quality requirements of the Indonesian National Standards for frozen bull semen, that is, sperm motility was more than 40% [15]. The dilution techniques did not affect sperm kinematic parameters (Table-1), but the SE value in the three-step dilution was quite high compared to those of one- and two-step techniques. This suggests that the quality of frozen semen diluted using a three-step technique varies greatly (i.e., the results are unstable).

### Sperm viability and membrane plasma integrity of Limousin bull semen frozen using various dilution techniques

Sperm viability values of each dilution technique showed different results. Sperm viability at one-step dilution was higher (p<0.05) than that of the three-step dilution (Table-2). There was no significant difference between the one-step and two-step techniques, or between the two- and three-step techniques (p>0.05). This study showed a decrease in the values of sperm viability that normally range between 18% and 23%. Sperm viability after freezing decreased around 20-35% [13]. The percentage of sperm viability in each dilution was good – sperm viability after thawing was >50%.

**Table-2:** Sperm viability of Limousin bull frozen semen in the skim milk-egg yolk extender on various dilution techniques.

Sperm parameters	Dilution techniques		

	One-step	Two-steps	Three-steps
Sperm viability of fresh semen (%)		94.07±0.76	
Sperm viability of frozen semen (%)	75.39±0.99^a^	72.34±1.37^ab^	70.16±1.73^b^
Decreased sperm viability (%)	18.68	21.73	23.91

Data show all mean±SEM. Means in lines with different superscripts is differ significantly at p<0.05. SEM=Standard error of means.

The integrity of the sperm plasma membrane from frozen semen used in this study also showed different results between dilution techniques. The highest value of plasma membrane integrity was in sperm diluted with the one-step technique and the lowest was in the three-step dilution (Table-3). The integrity of the plasma membrane is important because it can ensure sperm viability and protect the cytoplasm and DNA in the cells. The integrity of the membranes in the head of the sperm is also important because acrosomes located in the head contain enzymes that play an important role in the fertilization of eggs [19]. Damage to the plasma membrane in the tail, especially in the midpiece that contains mitochondria, causes inhibition of the production of adenosine triphosphate (ATP) as an energy source, rendering sperm immobile [20].

**Table-3:** Sperm plasma membrane integrity of Limousin bull frozen semen in the skim milk-egg yolk extender on various dilution techniques.

Sperm parameters	Dilution techniques		

	One-step	Two-steps	Three-steps
Sperm plasma membrane of fresh semen (%)		92.58±0.56	
Sperm plasma membrane of frozen semen (%)	73.60±0.81^a^	71.47±1.35^ab^	70.15±1.16^b^
Decreased sperm plasma membrane (%)	18.98	21.11	22.43

Data show all mean±SEM. Means in lines with different superscripts is differ significantly at p<0.05. SEM=Standard error of means.

The viability and integrity of the sperm plasma membrane in this study were good; presumably because of the suitable extender used, SMEY. Skim milk extender contains lactose, which can protect the sperm membrane. The addition of 10% egg yolk containing lecithin (phosphatidylserine choline) provides membrane coating and extracellular cryoprotectant, which help to maintain the normal configuration of the phospholipid bilayer of the membranes [21]. Molecular health of the sperm membranes is important for sperm functions such as motility and ability to fertilize the egg.

### Sperm DNA integrity of frozen Limousin bull semen subjected to various dilution techniques

**Figure-1:**
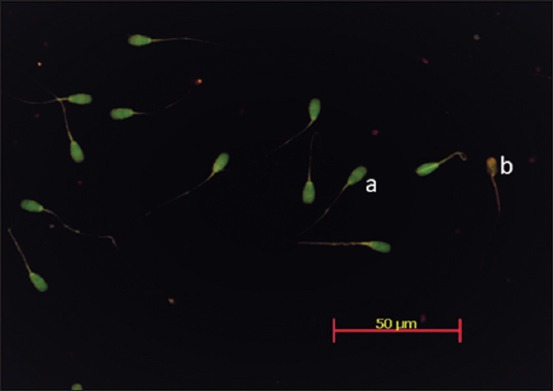
DNA fragmentation analysis using acridine orange staining. (a) Sperm with intact DNA. (b) Sperm with fragmented DNA.

Sperm DNA integrity can be inferred from the number of sperm with fragmented DNA. Fragmentation is the breaking of the DNA strands, known as DNA damage. The results showed that DNA fragmentation was low in sperm from each of the dilution techniques. DNA damage in one-, two-, and three-step dilutions was 0.33±0.23%, 0.46±0.17%, and 0.20±0.11%, respectively. It was classified by type as green (Intact) or orange-red (Fragmented) on differences in fluorescent color using *Acridine Orange* staining (Figure-1). There was no significant difference in DNA damage in sperm between dilution techniques (p>0.05). This shows that the frozen semen had low sperm DNA damage. Bochenek *et al*. [22] stated that fertility rates in bulls decline if DNA damage reaches 10%. The quality of sperm DNA is important for sperm physiology.

### Sperm MDA and AST levels in Limousin bulls’ frozen semen subjected to various dilution techniques

MDA level is an indicator of lipid peroxidation and is measured by the reaction of TBA. To determine the MDA concentration, the absorption readings of the pink supernatant were compared to standard curves of MDA produced by a hydrolysis reaction that was catalyzed by acids from 1,1,3,3-tetramethoxypropane. The results of the one- and three-step-dilution techniques showed similar MDA levels (p>0.05), which were 0.21±0.01 and 0.22±0.01 nmol/10^8^, respectively. Both were higher (p<0.05) as compared to the two-step-dilution (0.16±0.01 nmol/10^8^). The higher MDA levels indicate that lipid peroxidation occurred in sperm during freezing and thawing. Lipid peroxidation causes damage to the membranes of the sperm cells – the higher the cell damage, the lower the quality of the frozen semen [23].

The MDA levels in Limousin bulls’ frozen semen with SMEY extender have been reported [13] as 0.52±0.25 nmol/10^8^. The researchers used Limousin bulls aged 4-8 years from different AI Centers. The MDA levels can also differ depending on the breeds and extenders used. For example, MDA levels of Brangus–Simmental crossbred bull sperm in BioXcell^®^ extender can reach 1.94 nmol/10^8^ [24] and MDA levels of Merino sheep sperm in Tris-egg yolk extender were 5.55±0.55 nmol/10^8^ [25]. Sperm MDA levels, in this study, were low, suggesting that little lipid peroxidation occurred in the freezing and thawing process. Sperm cells have their own defense mechanisms through the antioxidant content of semen that plays a role in repairing the damage, but when it fails, an imbalance occurs between ROS and the antioxidant capacity of the semen. The imbalance between ROS and antioxidants is one of the most important factors contributing to the reduced quality of frozen semen. Low ROS levels are important for capacitation and some cell signaling pathways [26]. Conversely, increased ROS levels can cause lipid peroxidation [27] and reduce the quality of frozen semen.

**Figure-2:**
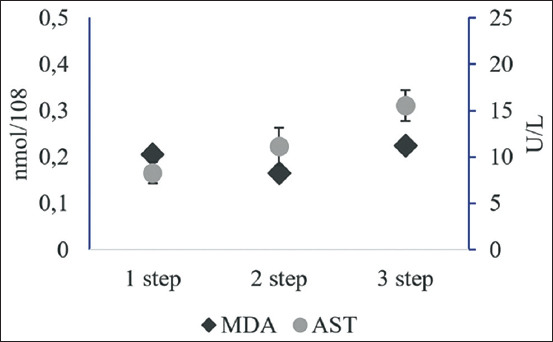
Malondialdehyde and aspartate aminotransferase levels of frozen Limousin bull semen in skim milk-egg yolk extender with various dilution techniques.

The AST enzyme levels in sperm cells indicate that cell damage is correlated with the integrity of the plasma membrane [28]. The AST enzyme levels in one-step diluted frozen semen were the lowest: 8.33±1.14 U/L (p<0.05). There was no significant difference between the enzyme levels of the one- and two-step dilutions (p>0.05). The three-step dilution showed the highest AST enzyme level: 15.55±1.65 U/L (Figure-2), but there was no significant difference between the two- and three-step dilutions.

The AST enzyme is important for metabolic processes that provide energy for cell survival, sperm motility, and fertility. AST enzyme activity in semen is a good indicator of quality and sperm membrane stability. Damage to the plasma membrane in the midpiece results in the release of AST enzymes into the semen plasma. If ATP production in the mitochondria of the sperm midpiece stops, the sperm cells cannot move, thereby causing a decrease in the quality of semen [29].

The two- and three-step-dilution techniques have several disadvantages. The first negative factor is the change in temperature when opening and closing the cooling cabinet during the addition of the second and third extenders. Unstable temperatures cause damage to sperm [30]. The second negative factor is the change in osmotic pressure induced by the additional extenders. Semen, in general, has an osmotic pressure between 280 and 300 mOsm [31]. Extender Part A (without glycerol) has an osmotic pressure between 300 and 400 mOsm [32]. Extender Part B (containing 16% glycerol) has an osmotic pressure of 1220-1280 mOsm [33]. We speculate that one addition of Part B extender (in two-step dilutions) causes the sperm to experience changes in temperature and osmotic pressure only once. Hence, we also propose that two additions of Part B extender (in three-step dilutions) cause changes in temperature and osmotic pressure twice. Changes in temperature and osmotic pressure cause stress to sperm, which could result in molecular changes, DNA damage, changes in the plasma membrane, and increased ROS. All of these result in decreased motility, which lowers overall sperm quality [9].

## Conclusion

The one-step dilution showed better viability, more intact plasma membranes, and lower AST levels than the three-step dilution. Based on our findings, we recommend the use of one- or two-step-dilution techniques for production of frozen bull semen in Indonesia.

## Authors’ Contributions

AAA and RIA conceptualized and designed the experiment, conducted the literature review, and wrote the first draft of the manuscript. TM and EMK collected data with CASA and DNA Integrity. BP and RIA edited and revised the draft of the manuscript. EM helped with interpretations of the results and revised and finalized the manuscript. All authors read and approved the final manuscript.
